# Outside-In Signalling Generated by a Constitutively Activated Integrin α_IIb_β_3_ Impairs Proplatelet Formation in Human Megakaryocytes

**DOI:** 10.1371/journal.pone.0034449

**Published:** 2012-04-23

**Authors:** Loredana Bury, Alessandro Malara, Paolo Gresele, Alessandra Balduini

**Affiliations:** 1 Division of Internal and Cardiovascular Medicine, Department of Internal Medicine, University of Perugia, Perugia, Italy; 2 Biotechnology Laboratories, Department of Biochemistry, University of Pavia, IRCCS San Matteo Foundation, Pavia, Italy; University of Birmingham, United Kingdom

## Abstract

**Background:**

The interaction of megakaryocytes with matrix proteins of the osteoblastic and vascular niche is essential for megakaryocyte maturation and proplatelet formation. Fibrinogen is present in the vascular niche and the fibrinogen receptor α_IIb_β_3_ is abundantly expressed on megakaryocytes, however the role of the interaction between fibrinogen and α_IIb_β_3_ in proplatelet formation in humans is not yet fully understood. We have recently reported a novel congenital macrothrombocytopenia associated with a heterozygous mutation of the β_3_ subunit of α_IIb_β_3_. The origin of thrombocytopenia in this condition remains unclear and this may represent an interesting natural model to get further insight into the role of the megakaryocyte fibrinogen receptor in megakaryopoiesis.

**Methodology/Principal Findings:**

Patients' peripheral blood CD45+ cells in culture were differentiated into primary megakaryocytes and their maturation, spreading on different extracellular matrix proteins, and proplatelet formation were analyzed. Megakaryocyte maturation was normal but proplatelet formation was severely impaired, with tips decreased in number and larger in size than those of controls. Moreover, megakaryocyte spreading on fibrinogen was abnormal, with 50% of spread cells showing disordered actin distribution and more evident focal adhesion points than stress fibres. Integrin α_IIb_β_3_ expression was reduced but the receptor was constitutively activated and a sustained, and substrate-independent, activation of proteins of the outside-in signalling was observed. In addition, platelet maturation from preplatelets was impaired.

**Conclusions/Significance:**

Our data show that constitutive activation of α_IIb_β_3_-mediated outside-in signalling in human megakaryocytes negatively influences proplatelet formation, leading to macrothombocytopenia.

## Introduction

Mature megakaryocytes (Mks) migrate to the vascular niche of the bone marrow where they convert the bulk of their cytoplasm into multiple long processes, called proplatelets, that protrude through the vascular endothelium into the sinusoid lumen to release platelets [1], [2]. Recently a new intermediate stage in platelet maturation has been described: preplatelets, discoid particles circulating in blood, larger than platelets, that reversibly convert into barbell-shaped proplatelets that in turn generate each two mature platelets after a fission event [3].

Very little is known about the role of specific bone marrow proteins in megakaryocyte differentiation and function. Fibrinogen was shown to be localized in the bone marrow sinusoids of mice and to be essential for proplatelet formation by binding to megakaryocyte α_IIb_β_3_ [4]. In fact, mouse megakaryocytes extend proplatelets when plated on fibrinogen, and treatment with α_IIb_β_3_ antagonists strikingly reduces the percentage of megakaryocytes developing proplatelets [4]. However, the interaction between integrin α_IIb_β_3_ and fibrinogen was shown to be essential for spreading but not for proplatelet formation by human megakaryocytes, and in fact while α_IIb_β_3_ antagonists almost abolished adhesion and spreading they did not cause any significant reduction of human proplatelet formation [5]. Indeed, Glanzmann Thrombasthenia (GT), a rare hereditary autosomal recessive bleeding disorder affecting the megakaryocytic lineage and due to quantitative and/or qualitative abnormalities of α_IIb_β_3_, is not associated with thrombocytopenia [6]. Therefore, the role of α_IIb_β_3_ in proplatelet formation in humans is still controversial.

We have recently described two families with a novel autosomal dominant hereditary mucocutaneous bleeding disorder with macrothrombocytopenia and defective platelet function associated with a heterozygous mutation (2134+1 G>C) of the ITGB3 gene, coding for the β_3_ subunit of α_IIb_β_3_ and producing a deletion (del647-686) of a large part of the β Tail Domain (βTD) [7], an extracellular domain of β_3_ involved in receptor activation [8]. This mutation, and in particular a mutation involving the β_3_ βTD domain, was never described before and it seemed of interest that it was associated with a reduced platelet number and altered platelet morphology.

Purpose of the present study was to analyse megakaryocyte maturation, spreading and proplatelet formation on fibrinogen, and other extracellular matrix proteins, in two patients with the Glanzmann variant macrothrombocytopenia associated with the β_3_ del647-686.

## Results

### Megakaryocyte differentiation and proplatelet formation

**Figure 1:**
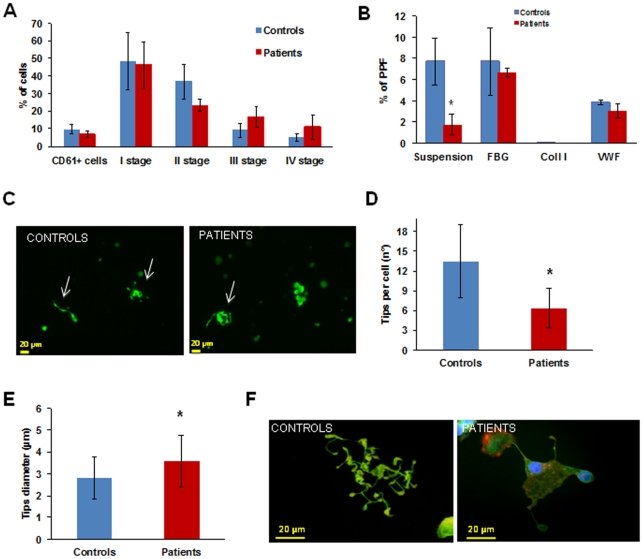
Megakaryocyte differentiation and proplatelet formation. (A) Megakaryocyte maturation stages of patients did not differ from those of controls. (B) Proplatelet formation (PPF) from patient megakaryocytes in suspension was drastically reduced. When megakaryocytes were plated on type I collagen proplatelet formation was absent, similar to controls, while on fibrinogen and von Willebrand factor the number of megakaryocytes extending proplatelets was normal. *p<0.05 vs control. (C) Representative pictures of proplatelet formation in suspension, in a control subject and a patient (20× magnification). Arrows indicate pro-platelets, only one developing proplatelet is evident in the patient sample. (D, E) Patient megakaryocytes extended a reduced number of proplatelets with abnormal characteristics: a spread shape with shorter than normal proplatelet shafts and tips significantly decreased in number and larger in size than those of controls. *p<0.05 vs control. (F) Representative images of megakaryocytes from patients and controls releasing proplatelets upon adhesion to fibrinogen.

The percentage of CD45+ cells differentiated into megakaryocytes was comparable in patients and controls (7±1.8% vs 9.9±2.7%, respectively, p = ns). Megakaryocyte maturation profiles, classified according to standard criteria [9], were not significantly different between patients and controls, indicating that del647-686 of â_3_ integrin does not affect the differentiation or maturation of megakaryocytes (**Figure 1A**).

Proplatelets formed by megakaryocytes in suspension were instead reduced, with only 1.8±1.0% of patient megakaryocytes extending proplatelets vs 7.7±2.2% of controls after 16 hours (p<0.05) (**Figure 1B**), but with a normal morphology. Defective proplatelet formation was confirmed in experiments with a longer incubation time (24 hours) (data not shown).

On the contrary, when megakaryocytes were plated on fibrinogen proplatelets were numerically comparable to those of controls (7.7±3.2% vs 6.7±0.4% n = 3, p = ns), but presented important structural alterations, with megakaryocytes showing a spread shape, shorter proplatelet shafts and tips significantly decreased in number and larger in size than those of controls (**Figure 1C, 1D and 1E**). Interestingly, while pro-platelet formation usually starts from one pole of the megakaryocyte and rapidly leads to the conversion of the entire cytoplasm into proplatelets [2] **,** in our patients pro-platelet formation started at multiple poles of the megakaryocyte cell body (**Figure 1E** **, right**)**.** Finally, proplatelet formation from patient's megakaryocytes incubated with type I collagen or Von Willebrand factor was not different from that of control megakaryocytes [10**]**–[****12] (on type I collagen: 0.1±0.2% vs 0.1±0.2%, p = ns; on VWF 3.0±0.6% vs 3.8±0.2%, p = ns) (**Figure 1B**).

### Megakaryocyte spreading

**Figure 2:**
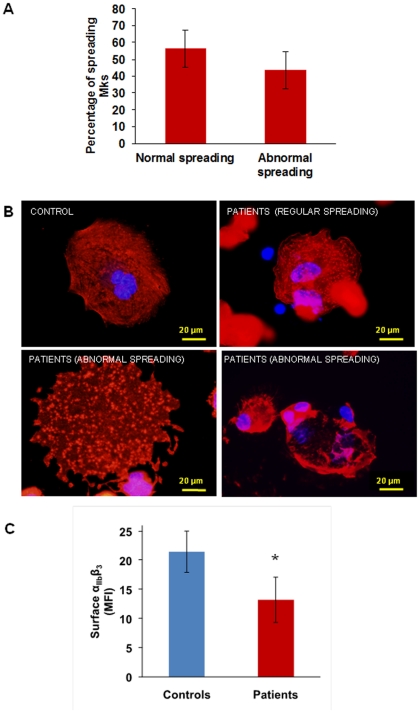
Megakaryocyte spreading on fibrinogen and α_IIb_β_3_ expression. (A) and (B) When plated on fibrinogen two populations of megakaryocytes are visible: half of the population spread regularly, while half showed abnormal spreading, with nuclei displayed towards cell periphery, a disordered distribution of actin and focal adhesion points more evident than stress fibres. (C) Flow cytometry showed decreased expression of α_IIb_β_3_ on the surface of patient's megakaryocytes as compared with control megakaryocytes. *p<0.05 vs control.

Megakaryocyte spreading on type I collagen was similar in patients and controls (15.4% vs 18.6±3.3% of the total adhering population, p = ns) as well as spreading on Von Willebrand factor (5.2% vs 5.9±1.3% of the total adhering population, p = ns). On the contrary, spreading on fibrinogen was increased: 31.7±5.6% of the total population of adhering megakaryocytes compared to 10±2% of controls (p<0.05). Two different populations were detectable among patient megakaryocytes: one spreading normally (56.4 ±11% of the total spread population), and the other abnormally (43.5±11% of the total spread population) (**Figure 2A**).

Abnormally spread megakaryocytes showed nuclei displaced towards cell periphery, a disordered distribution of actin and focal adhesion points more evident than stress fibres (**Figure 2B** **lower panels**). Normally spread megakaryocytes, instead, showed central nuclei and an ordered organization of actin in stress fibres and focal adhesion points, similar to controls (**Figure 2B** **upper panels**).

### α_IIb_β_3_ expression and activation

Integrin α_IIb_β_3_ was significantly less expressed on the surface of patients' megakaryocytes than on that of control megakaryocytes (mean fluorescence intensity: 13.2±2.1 vs 21.5±2.2%, respectively, p<0.05) (**Figure 2C**), in accordance with what we previously observed with the patients' platelets [7].

As β_3_ integrin is also a subunit of the α_V_β_3_ receptor (CD51/61), we measured α_V_β_3_ by flow cytometry and we found that its expression was comparable between patients and controls, both in platelets (patients 4.8±0.6% vs controls 4.8±0.4%, p = ns) and in megakaryocytes (patients 13±1.1% vs controls 10.7±1.6%, p = ns) (data not shown).

**Figure 3:**
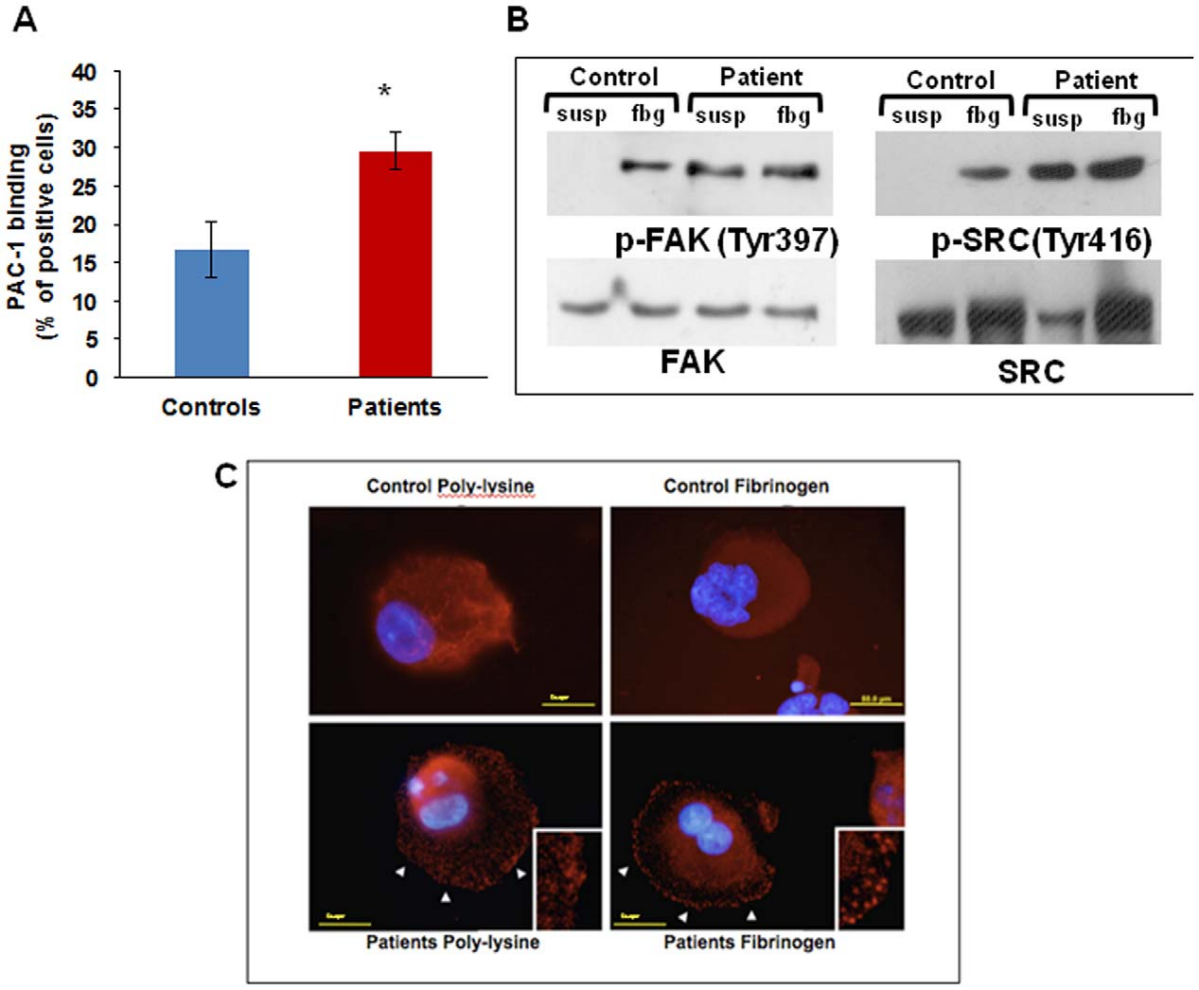
Integrin α_IIb_β_3_ activation and outside-in signalling. (A) Flow cytometry analysis of PAC-1 binding to resting megakaryocytes is significantly increased in patients as compared with controls. *p<0.05 vs control. (B) Western blotting showed Src and FAK phosphorylation in patient megakaryocytes in suspension as well as after adhesion onto fibrinogen. (C) Differently from control cells (upper panels), patient megakaryocytes showed FAK clustering already after 1 hour of adhesion onto fibrinogen, and also in suspension (lower panels).

To study α_IIb_β_3_ receptor activation we measured the binding of PAC-1, a monoclonal antibody that binds only to activated α_IIb_β_3_, to un-stimulated megakaryocytes: 29.5±0.9% of patients' megakaryocytes bound PAC1 vs 16.7±3.6% of control megakaryocytes (**Figure 3A**), showing constitutive activation of α_IIb_β_3_ integrin in patients' megakaryocytes.

We therefore assessed α_IIb_β_3_-triggered outside-in signalling by measuring the phosphorylation of FAK and Src after adhesion to fibrinogen by western blotting [13] **.**

Src and FAK were phosphorylated in patients' megakaryocytes also in suspension while in controls phosphorylation was observed only upon adhesion to fibrinogen (**Figure 3B**).

We also assessed FAK clustering at immunofluorescence: clustering was clearly evident in patients' megakaryocytes already one hour after plating on fibrinogen (**Figure 3C right panel**)**,** while with control megakaryocytes it was evident only after 3 hours. Moreover, FAK clusters were observed in patients' megakaryocytes in suspension, differently from controls were they were evident only upon contact with fibrinogen (**Figure 3C left panel**), consistently with constitutive activation of α_IIb_β_3_.

### Conversion of preplatelets into platelets

**Figure 4:**
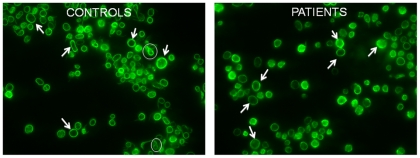
Conversion of preplatelets into mature platelets. In patient's peripheral blood less “figure 8” shapes are present. Two “figure 8” shapes are circled in white in control blood (left), while no “figure 8” shapes are visible in this picture of patient peripheral blood (right). Arrows show examples of preplatelets.

Very recently a new intermediate form between proplatelets and platelets, the preplatelet, was described [3]. A preplatelet can reversibly convert into a barbell-shaped proplatelet and generate two platelets passing through a “figure 8” structure. We therefore counted “figure 8” structures in platelet rich plasma (PRP) and we observed a significantly lower percentage of them in patients' PRP as compared to controls (0.5±0.7% vs 2.1±1.2% respectively, p<0.05) (**Figure 4**).

## Discussion

Extracellular proteins play an important role in megakaryopoiesis and platelet formation by interacting with their receptors on megakaryocytes [5]. In particular, the vascular niche is enriched in fibrinogen and von Willebrand factor which drive the late phases of megakaryopoiesis and allow proplatelet formation and platelet release [4], [5].

Here we show that two patients with a variant form of Glanzmann Thrombasthenia (GT) associated with macrothrombocytopenia, which is not normally present in GT, due to a partial deletion of integrin β_3_ [7] have megakaryocytes that, despite normal differentiation, fail to extend proplatelets in suspension, form abnormal proplatelets on fibrinogen, and show reduced preplatelet maturation.

Patient megakaryocytes expressed significantly less α_IIb_β_3_ on their surface, as already seen with platelets [7], but this was constitutively activated, as shown by PAC-1 binding under resting conditions, by faster spreading upon contact with fibrinogen and by FAK clustering and Src and FAK phosphorylation in suspension. A constitutively activated α_IIb_β_3_ in our patient megakaryocytes is consistent with a deletion involving the βTD, a portion of α_IIb_β_3_ with a key role in maintaining the receptor in its low affinity conformation, [8] and with our previous observation that CHO cells expressing the β_3_ del647-686 mutation bind fibrinogen without the need of activation [Bury L, Cecchetti L, Giannini S, Corazzi T, Appolloni V, et al. (2010) Impact of a novel integrin β_3_ mutation (del647-686), associated with a Glanzmann's variant hereditary platelet defect, on GPIIb/IIIa expression and signalling. Blood Transfus 8: OC067].

The expression of α_V_β_3_, the receptor for vitronectin, was instead normal probably due to the structural differences between α_IIb_ and α_V_ in their calf2 domains [8], [14], the domain interacting with βTD.

Spreading on fibrinogen showed a peculiar pattern, with half of the population spreading normally, and half showing abnormal spreading, similar to patients’ platelets [7] suggesting that a preferential segregation of the mutant β_3_ subunits in clusters occurs in some cells but not in others upon ligand binding to α_IIb_β_3_ [15].

Megakaryocyte spreading and proplatelet formation on Von Willebrand Factor (VWF) were instead normal, which may be unexpected because VWF is a ligand for α_IIb_β_3_. Given that in platelets [16] and in α_IIb_β_3_ expressing CHO cells [17] the α_IIb_β_3_-VWF interaction occurs only after integrin activation and that signalling through GPIb-IX-V activates α_IIb_β_3_ [18], it is conceivable that in megakaryocytes contact with VWF GPIb-IX-V activates α_IIb_β_3_ that, in turn, promotes spreading. If this were the case, in our patients a constitutively activated α_IIb_β_3_ would not perturb VWF-mediated megakaryocyte spreading while it would affect fibrinogen-mediated spreading, where this activation is not required.

Also proplatelet formation on fibrinogen was abnormal in our patients, with a reduced number of proplatelets with enlarged tips, in agreement with two recent reports describing patients with gain-of-function mutations of α_IIb_β_3_ associated with thrombocytopenia, one at the cytoplasmic tail of β_3_ [19] and the second at the cytoplasmic domain of α_IIb_ [20]. Differently from these reports, that demonstrated constitutive α_IIb_β_3_ activation only in cells transfected with the mutant integrin [19], [20], our study shows for the first time a constitutively activated α_IIb_β_3_ in patients' megakaryocytes. Altogether these observations show that an absent α_IIb_β_3_ is less disruptive to thrombopoiesis than a hyper-active receptor, suggesting that outside-in signalling must be “switched off” during platelet production.

Our data also suggest that actin remodelling is critical in the late phases of fibrinogen-induced proplatelet formation. In fact, ligand-binding to α_IIb_β_3_ induces the activation of c-Src, normally associated with the cytoplasmic tail of β_3_ in resting megakaryocytes, and then FAK activation that in turn stimulates actin remodelling leading to cell spreading [21]. A constitutively activated FAK-Src signalling, as observed in our patients' megakaryocytes, leads to permanent actin polymerization and this may cause abnormal proplatelet formation, as shown by treatment of megakaryocytes with an inhibitor of actin assembly, cytochalasin [22], or by macrothrombocytopenia in mice genetically deficient of ADF or cofilin, two proteins involved in actin depolymerization [23].

Recently it has been shown that in the late maturation steps leading to the formation of platelets, a malleable cytoplasm is essential for the passage from preplatelets, large, oval-shaped circulating platelet precursors, into barbell-shapes, by the twisting of their microtubule cytoskeleton around their centre to yield “figure 8” structures, and finally into two individual platelets [3]. It is therefore conceivable that a rigid, constitutively activated actin network may hinder proplatelet formation and lead to the formation of platelets of an abnormal size, compatible with the reduced maturation of preplatelets into platelets observed in our patients' blood.

In conclusion, impaired proplatelet formation from megakaryocytes, together with a normal number of reticulated platelets excluding enhanced platelet destruction, lead us to conclude that macrothrombocytopenia in our patients is due to defective platelet formation. Our results show that constitutive activation of α_IIb_β_3_-mediated outside-in signalling in human megakaryocytes negatively influences proplatelet formation and open new perspectives in the study of the role of the α_IIb_β_3_–fibrinogen axis in platelet formation and related diseases.

## Materials and Methods

### Cell culture and immunofluorescence

CD45+ cells were separated from peripheral blood of the patients and healthy controls and cultured as previously described [5], [11], [24].

All subjects gave written informed consent to the study, which was approved by the Committee on Bioethics of the University of Perugia.

Megakaryocyte differentiation was evaluated at day 14 of culture on cells (1×10^5^) cytospun on poly-L-lysine-coated glass coverslips (Sigma-Aldrich, Milan, Italy) and stained with an anti CD41 antibody or with May Grunwald Giemsa, as previously described [10], [25]. CD41 positive cells were classified according to dimensions and nuclear configuration.

To evaluate proplatelet formation and megakaryocyte spreading onto adhesive substrates megakaryocytes at day 14 of culture were separated on a BSA gradient (3–4%), plated onto glass coverslips coated with 100 µg/ml human fibrinogen (Sigma-Aldrich, St. Louis, MO, USA), 10 µg/ml VWF-rich concentrate (Haemate P; Aventis-Behring, Milan, Italy) or 25 µg/ml type I collagen from bovine tendon (kind gift of prof M.E. Tira, University of Pavia) in 24-well plates (1×10^5^ cells *per* well), and allowed to adhere for 16 h at 37°C and 5% CO_2_. To evaluate proplatelet formation in suspension, megakaryocytes were seeded in 24 well plates and incubated for 16 h at 37°C and 5% CO_2_.

Samples were then analyzed by immunofluorescence as previously described [5] **.** Analysis was performed on 20 different fields for each sample. For tips diameter and number measurements, at least one hundred tips per sample were measured.

Proplatelet formation and megakaryocyte spreading onto different substrates were calculated as the percentage of proplatelets or spread megakaryocytes over the total megakaryocyte population adhering to the substrate. Normal and abnormal spreading on fibrinogen are expressed as the percentage of normal or abnormal shapes out of the total population of spreading megakaryocytes.

FAK phosphorylation by immunofluorescence was assessed on megakaryocytes plated for 1 hour onto fibrinogen or poly-L-lysine using an anti phospho-FAK (Tyr 397) antibody (Cell Signalling, Danvers, MA, USA).

### Flow cytometry

CD41 expression was analyzed by flow cytometry after incubation of Mks for 30 minutes with the FITC-conjugated mAb anti-CD41 clone P2 (Beckman Coulter, Miami, FL, USA). CD51 expression was analyzed incubating Mks and platelets for 30 minutes with a FITC-conjugated anti-CD51 mAb (Immunotech, Marseille, France). To measure α_IIb_β_3_ activation Mks were incubated for 30 minutes with the PAC-1 FITC mAb (BD Biosciences, Milan, Italy), that recognizes activated α_IIb_β_3_. PAC-1 binding is expressed as the percentage of megakaryocytes that bind PAC-1 out of the total number of CD41-expressing cells.

For every mAb an isotypic antibody was used as a negative control. Samples were analysed in an EPICS XL-MCL flow cytometer (Beckman Coulter, Miami, FL, USA), equipped with an argon laser operating at 488 nm [26].

### SDS page and Western Blotting

Patient and control megakaryocytes were plated for 3 h at 37°C on 12-well plates pre-coated with 100 µg/ml of purified human fibrinogen or 1% BSA. Cells were then washed twice with PBS and lysed with lysis buffer (40 mM Tris- HCl, 0.3 M NaCl, 1 mM EDTA, 1 mM NaF, 1 mM Na_3_VO_4_, 10 μl NP-40, 10 µg/ml leupetin/pepstatin).

An equal amount of proteins were resolved by 8% SDS-polyacrylamide gel electrophoresis (PAGE) and transferred onto nitrocellulose. Membranes were probed with a rabbit anti-phospho–FAK (Tyr 397) or an anti-FAK MoAb, with a rabbit anti-phospho-Src (Tyr416) or an anti-Src (Cell Signalling Technology, Danvers, MA) MoAb and immunoreactive bands were detected using peroxidase-conjugated secondary antibodies and chemiluminescence detection.

### Preplatelet “figure 8” counting

Patient and control blood was centrifuged at 100 g for 20 minutes to obtain PRP; 10^6^ platelets were then cytospun on poly-L-lysine-coated glass coverslips (Sigma-Aldrich, St. Louis, MO, USA), fixed with 4% PFA for 20 minutes, permeabilized with 0.1% Triton-X for 5 minutes, blocked with 3% BSA for 2 hours, stained with an anti-β1 tubulin antibody (a kind gift of professor Joseph Italiano, Boston, USA) and then with a secondary antibody conjugated with Alexa Fluor 488 (Invitrogen, Life Technologies, Grand Island, NY, USA). Specimens were mounted in Mowiol (Calbiochem, Merck, Darmstadt, Germany) and analyzed through a Carl Zeiss Axio Observer. A1 fluorescence microscope, using a 100X/1,4 Plan-Apochromat oil-immersion objective.

“figure 8” preplatelets were counted as the percentage of “figure 8” shapes over the total number of β1 tubulin-positive elements plated on the slide; the analysis was performed on 20 different fields for each sample.

### Statistic analysis

Data are presented as means ± SD. T test for unpaired data or two way ANOVA were used to analyze data, with a significant difference set at p<0.05.
